# Characterization of Biomaterials by Soft X-Ray Spectromicroscopy

**DOI:** 10.3390/ma3073911

**Published:** 2010-07-06

**Authors:** Bonnie O. Leung, John L. Brash, Adam P. Hitchcock

**Affiliations:** 1 Department of Chemistry & Chemical Biology, McMaster University, Hamilton, ON, L8S 4M, Canada; E-Mail: leungbo@mcmaster.ca (B.O.L); 2 School of Biomedical Engineering, McMaster University, Hamilton, ON, L8S4M1, Canada; E-Mail: brashjl@mcmaster.ca (J.L.B.)

**Keywords:** photoemission electron microscopy, X-PEEM, NEXAFS, scanning transmission X-ray microscopy, STXM, mapping, protein adsorption, human serum albumin, fibrinogen, polymers, blend, topography

## Abstract

Synchrotron-based soft X-ray spectromicroscopy techniques are emerging as useful tools to characterize potentially biocompatible materials and to probe protein interactions with model biomaterial surfaces. Simultaneous quantitative chemical analysis of the near surface region of the candidate biomaterial, and adsorbed proteins, peptides or other biological species can be obtained at high spatial resolution via scanning transmission X-ray microscopy (STXM) and X-ray photoemission electron microscopy (X-PEEM). Both techniques use near-edge X-ray absorption fine structure (NEXAFS) spectral contrast for chemical identification and quantitation. The capabilities of STXM and X-PEEM for the analysis of biomaterials are reviewed and illustrated by three recent studies: (1) characterization of hydrophobic surfaces, including adsorption of fibrinogen (Fg) or human serum albumin (HSA) to hydrophobic polymeric thin films, (2) studies of HSA adsorption to biodegradable or potentially biocompatible polymers, and (3) studies of biomaterials under fully hydrated conditions. Other recent applications of STXM and X-PEEM to biomaterials are also reviewed.

## 1. Introduction

Upon implantation in biological tissue or first contact with blood, materials are immediately coated with a layer of proteins. The properties of this initial protein layer can have a very strong effect on biocompatibility [1]. Characterization of the surfaces of biomaterials and their interaction with relevant proteins, peptides or other biologically active species can aid in determining and understanding biocompatibility. The research reviewed here speaks to one of the broad objectives of modern biomaterials research, namely understanding and controlling protein surface interactions. Control in this context includes both complete prevention or minimization of adsorption (protein resistance, antifouling) as well as promotion of adsorption of one specific protein from the contacting biological tissue or fluid. Maintenance of protein bioactivity is also required in the latter case.

Control at the level of localization of protein on surfaces that are heterogeneous (chemical, physical properties), has received less attention, although its relevance to aspects such as surface patterning (e.g., in biochip applications) is clear. Several studies have been reported on gradient surfaces where the properties vary gradually along one dimension. An example is the work of Elwing *et al*. on hydrophobicity gradients [2] which showed that in general adsorption of proteins was stronger at the hydrophobic end of the gradient. More recently Ekblad *et al*. studied adsorption on surfaces having a gradient in electrical charge [3]. Adsorption from various protein solutions (including plasma) was shown to be a minimum at the composition corresponding to zero net charge.

Our work using NEXAFS based methods as discussed in this review, also addresses questions regarding localization but at a more detailed and fundamental level. We ask: “*given a surface that is chemically and/or physically heterogeneous, eg a microphase separated block copolymer system, what localization preferences does an adsorbing protein show, in both a thermodynamic and a kinetic sense*?” In particular we have been interested in the early stages of adsorption when the surface is essentially empty of protein.

The distribution of the chemical components of the substrate or selective adsorption of bio-components to the surface is not always at a size scale which can be monitored by traditional microscopy or chemical analysis techniques. For example, Fourier transform infrared spectroscopy (FTIR) [4], surface plasmon resonance (SPR) [5,6,7], ellipsometry [8], quartz crystal microbalance (QCM) [9], and radiolabeling [10] provide good analytical sensitivity with limited spatial resolution, while atomic force microscopy (AFM) [11,12] and transmission electron microscopy (TEM) [13] offer sub-nm spatial resolution but limited chemical recognition. Synchrotron-based spectromicroscopy techniques such as X-ray photoemission electron microscopy (X-PEEM) [14,15] and scanning transmission X-ray microscopy (STXM) [16,17,18], which are based on near-edge X-ray absorption fine structure (NEXAFS) spectroscopy, afford a suitable balance of spatial resolution and chemical sensitivity, with quantitative chemical characterization at the C 1s edge achieving detection limits in the part per thousand range with good spatial resolution (80 nm X-PEEM, 30 nm STXM). A comprehensive review of soft X-ray spectromicroscopy applications to polymers and other soft materials such as biological species was recently published [19].

Other works have been published on protein adsorption to phase separated polymer surfaces. For example, Sengonul *et al*. investigated the adsorption of wild type and modified ferritin on a homopolymer blend of poly(desaminotyrosyl tyrosine dodecyl ester carbonate) (PDTD) and poly( -caprolactone) (PCL) [20]. The transmission electron micrographs showed preferential adsorption of modified ferritin to the PCL domains and of wild ferritin to the PDTD. Seo *et al*. investigated phase-separated block copolymer surfaces based on poly(2-methacryloyloxyethyl phosphorylcholine (MPC)) (PMPC) and poly(dimethylsiloxane) (PDMS) by TEM and AFM [21]. The hydrophobic PDMS domains adsorbed protein preferentially and cell adhesion increased with increasing PDMS domain size of the block copolymers in serum. Using our NEXAFS spectromicroscopy methods, the chemical selectivity and spatial resolution is such that one can pinpoint not only domain localization but inter-domainal localization as well.

Since STXM is a photon-in, photon-out technique, soft X-rays in the “water window” (200–520 eV) have enough energy to penetrate through 1–2 μm of water; thus, fully hydrated samples can be analyzed. This is especially important for protein adsorption studies, where water is essential for the native protein structure [22,23]. Soft X-ray PEEM is an ultra high vacuum (UHV) technique, which is highly surface sensitive, probing only the top 10 nm of the film surface [19]. This surface sensitivity is a major advantage for visualizing and quantitatively mapping proteins to surfaces, especially when the composition of the surface is different from the bulk. Furthermore, since most biological reactions occur at interfaces and surfaces [1], analytical techniques with surface sensitivity are particularly important for elucidating the mechanisms of these reactions.

Recently, a number of model biomaterials have been examined with X-ray spectromicroscopy with an emphasis on three major areas: (1) characterization of hydrophobic materials and protein interactions with these surfaces, (2) protein adsorption to biodegradable or biocompatible polymers, and (3) evaluation of biomaterials under hydrated conditions.

PS/PMMA surfaces have been optimized for phase segregation in the 1–2 μm range as a model biomaterial with hydrophobic non-polar (PS) and polar (PMMA) domains [24]. Fibrinogen (Fg), a protein with a major role in blood coagulation and thrombosis [25], was adsorbed to PS/PMMA from buffer and DDI water and compared to ^125^I radiolabeling results [26]. Human serum albumin (HSA), the most abundant plasma protein and a carrier of fatty acids [27], was adsorbed to PS/PMMA and examined by X-PEEM with respect to protein concentration [28], adsorption time [28], pH [29] and protein-peptide competitive adsorption [30].

PS-polylactide (PLA) [31] and chitosan-PMMA blend surfaces were optimized for protein adsorption studies by analyzing the phase segregation at the surfaces of these materials by X-PEEM. HSA adsorption to PS-PLA was studied as a function of ionic strength of the adsorption environment. It was demonstrated that mapping of the HSA at the phase segregated PS-PLA surface was enhanced when the same area was investigated with multi-edge (C 1s and N 1s) mapping [32].

Polyethylene oxide (PEO) crosslinked with pentaerythritol triacrylate (PETA) at varying PETA concentrations was also examined by these methods. It was found that PETA segregated to the substrate-air surface. Furthermore, HSA adsorption increased with increasing PETA concentration, showing that the protein resistance of PEO was reduced with increasing concentrations of PETA crosslinker [33].

X-PEEM has also been used to examine fluorocarbon coatings on stents to evaluate the effectiveness of the coating to prevent potential inflammatory transition metals such as nickel, molybdenum and chromium from leaching into the blood [34]. In that study, X-PEEM maps acquired for Cr, Fe, C and F gave insight into the origins of pinhole defects which could compromise the effectiveness of the coatings.

Fibrinogen adsorption to polymeric nanoparticles embedded in polyurethane [35] and HSA adsorption to PS/PMMA were examined under fully hydrated conditions [36] using STXM with the {protein, water/buffer} solution and the polymer substrate sandwiched between two X-ray transparent silicon nitride (Si_3_N_4_) windows. Furthermore, electrolyte-induced de-swelling behavior of microgels (potential drug carriers) induced by changing pH was imaged with STXM in a wet cell [37].

Many other NEXAFS spectroscopy studies of biomaterials or biomolecules have been described in the literature. Among these we note: Fg adsorption to single walled carbon nanotube films [38], bio-selective polymer brushes [39], ultra-thin films of histidine on gold [40], and characterization of the speciation, orientation and conformation of peptides, protein and DNA on various surfaces [41,42,43,44,45,46,47]. However, the intent of this review is to examine specific examples of spatially resolved model biomaterials or protein adsorption to biologically relevant surfaces using synchrotron-based X-ray microscopy in either full field imaging (X-PEEM) or scanning microprobe (STXM) modes, combined with NEXAFS spectroscopy. In particular, the results of these studies are primarily intended to provide insight into the interaction of blood and blood components with randomly patterned polymers; and are part of an on-going effort to develop biomaterials with improved biocompatibility in blood contact applications.

## 2. Spectromicroscopy Background

### 2.1. X-ray Photoemission Electron Microscopy (X-PEEM)

**Figure 1:**
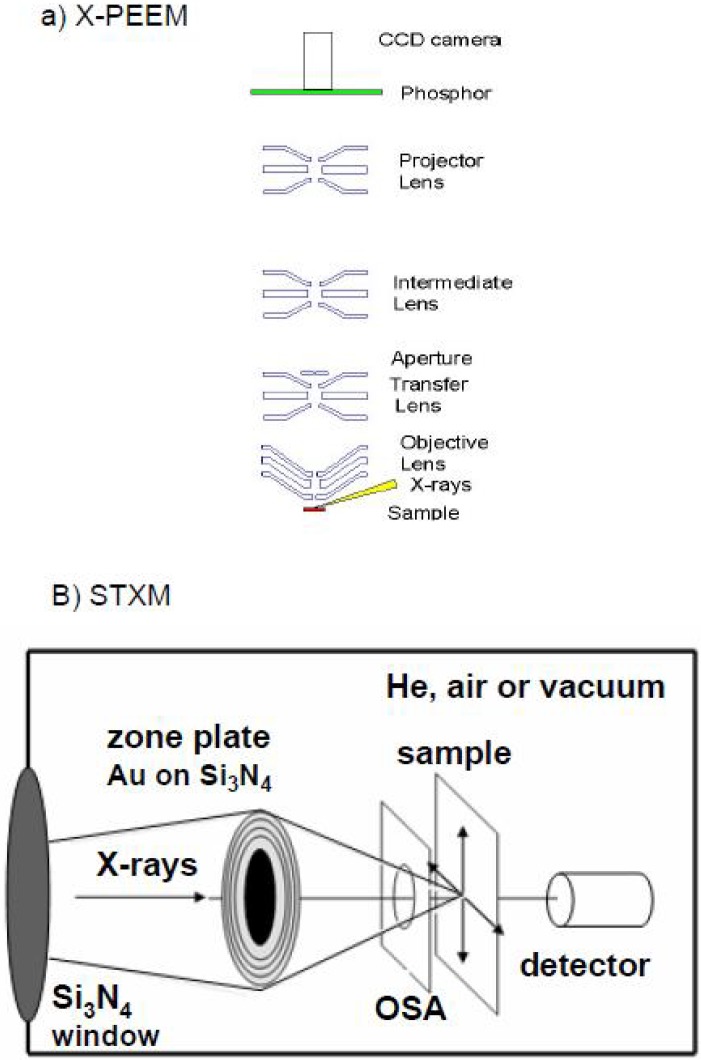
Schematic of the optics in (a) ALS PEEM-2 (figure courtesy of Andreas Scholl, ALS, LBNL.) and (b) STXM.

The X-PEEM experiments described in this review were performed at the Advanced Light Source (ALS) on bend magnet beamline 7.3.1 (PEEM-2) [48] or at the Canadian Light Source (CLS) on the spherical grating monochromator beamline 11ID1 or the spectromicroscopy beamline, 10ID1. At the ALS, a beam of left or right elliptically polarized X-rays of approximately 30 μm diameter strikes the sample at 15° under ultrahigh vacuum (UHV) conditions causing the ejection of photoelectrons and secondary electrons. The electrons are accelerated by a strong electric field (14–18 keV over 1–2 mm) into an electrostatic imaging column where the distribution of electrons is magnified by electrostatic lenses (Figure 1a). The electrons impinge a phosphor screen and the resulting light image is captured by a CCD camera. A 100 nm thick titanium filter is used to remove second order light and a shutter with a 0.1 s response time is used to block the X-ray beam except when acquiring images. In the Elmitec PEEM instrument used at the CLS, magnetic lenses and an image intensifier are used, but otherwise the optical principles are similar. X-PEEM is a surface sensitive technique with a sampling depth (1/e) of 4 nm for polymers with an integrated sampling depth of 10 nm [49]. X-PEEM requires conducting samples to replenish the charges removed by photoemission, and this avoids sample charging. Since polymeric biomaterials are insulators it is essential to use very thin (typically below 50 nm, although polymer films as thick as 250 nm have been successfully studied–see sec. 3.2.3) films of the polymer on a reasonably conducting and ultra-flat substrate (undoped Si is suitable).

### 2.2. Scanning Transmission X-ray Microscopy (STXM)

All STXM experiments were performed at the ALS on beamline 5.3.2. Comprehensive descriptions of the STXM beamline [50,51] and optics [52] are provided elsewhere. Briefly, X-rays from a bend magnet are focused by a toroidal mirror and a spherical grating monochromator selects X-rays of a specific energy with the coherence defined by one entrance and two orthogonal exit slits. The X-rays are focused by a Fresnel zone plate to a spot size of 25 nm (Figure 1b). An order sorting aperture (OSA) with a central stop blocks zero-order light. The sample is raster scanned in the x-y direction and transmitted X-rays are detected by a phosphor and converted to visible light pulses which are counted by a photomultiplier.

Very thin samples are required for STXM, with the optimum thickness at the C1s edge around 100 nm. For STXM, samples are usually spun-cast or microtomed. Typically, polymer films are spun cast onto mica and then floated onto a silicon nitride (Si_3_N_4_) window for imaging. For hydrated wet cells, the sample is sandwiched between two Si_3_N_4_ or polyimide windows with samples having < 2 μm of water leaving sufficient accessible optical density for studies at the C 1s edge.

### 2.3. Data Analysis

Data analyses for both X-PEEM and STXM were performed with the aXis2000 software package [53]. C 1s and N 1s image sequences (or stacks [54]) were aligned, normalized to the ring current and for X-PEEM, divided by the I_0_ spectrum collected from a clean HF-etched Si(111) chip, while for STXM, the images were converted into optical density (OD). The near edge X-ray absorption fine structure (NEXAFS) spectrum obtained at each pixel of the stack are fit to known reference spectra via singular value decomposition (SVD), which is an optimized method of least squares analysis [55,56]. The fit coefficients from the SVD analysis result in component maps which show the spatial distribution of each inputted reference spectrum. For X-PEEM, skewed illumination was corrected by dividing the stack with a heavily smoothed image of the sum of all the component maps. The component maps were then adjusted by a scale factor such that the total average thickness of all the components summed to 10 nm, the approximate sampling depth of X-PEEM.

To obtain quantitative results, a threshold mask was applied to each component map to obtain pixels corresponding to a specific region (e.g., polystyrene, poly(methyl methacrylate) or the inter-domainal interface). The average NEXAFS spectrum found for each region was then fit with reference spectra that were normalized to the theoretical intensity for 1 nm of the bulk material.

Radiation damage is a concern when examining polymeric and biological materials with STXM and X-PEEM, and measures were taken to ensure the minimum possible radiation exposure that would provide adequate statistical precision. In the STXM and X-PEEM, a fast shutter (0.5 ms for STXM, 0.1 s for PEEM) was used to blank out the beam except during data acquisition. To minimize radiation damage images are collected at only twenty-five to forty energies. X-PEEM analysis is also complicated by the requirement for conducting samples; polymer films thicker than ~250 nm usually result in charging.

## 3. Results and Discussion

### 3.1. Spectromicroscopy Applications

#### 3.1.1. Polystyrene-Poly(methyl methacrylate) (PS-PMMA)

##### 3.1.1.1. Optimization of PS-PMMA blend surface

Since PS and PMMA are immiscible polymers, their blends phase segregate in the bulk [57] and at the surface [58]. The resulting system of PS-rich and PMMA-rich domains serves as a suitable model for a polymeric biomaterial with regions having different surface polarities and hydrophobicities, which may drive selective protein adsorption.

**Figure 2:**
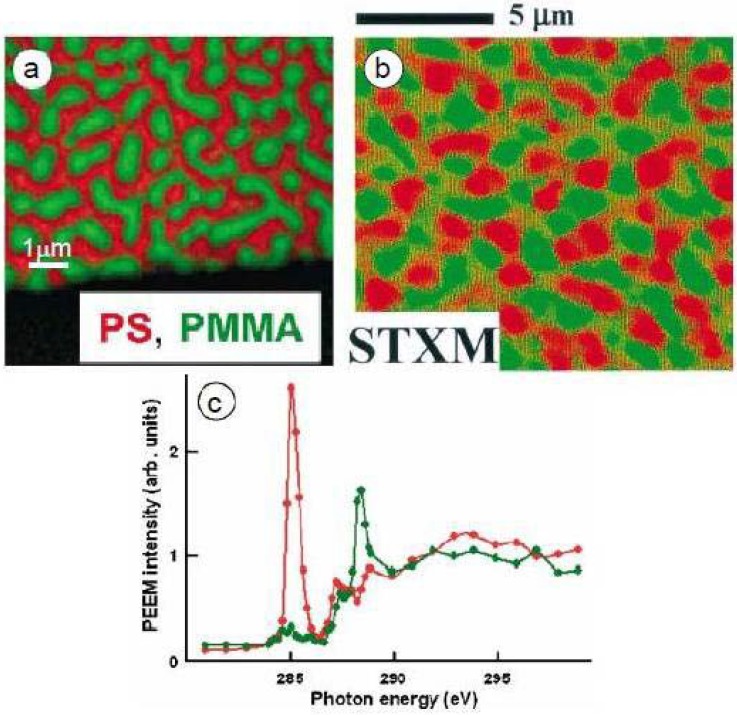
Comparison of (a) X-PEEM and (b) STXM imaging for PS/PMMA 30:70 w/w. (c) C 1s NEXAFS spectra of PS (red) and PMMA (green) recorded in PEEM2 [24].

Blends of PS/PMMA with varying molecular weights and different mass ratios were combined and spun cast onto native oxide silicon or silicon nitride windows for X-PEEM or STXM analysis, respectively [24]. Similar PS-rich surfaces were obtained for PS/PMMA blend ratios of 90/10, 66/33, and 30/70 w/w using high molecular weight polymers (PS = 1M, PMMA 300K), which suggests that the thin films may not be in thermodynamic equilibrium [24]. X-PEEM imaging of the PS/PMMA 30/70 film annealed at 160 °C (2 h-18 h) gave slightly different results compared to STXM (Figure 2a). X-PEEM, which samples the top 10 nm of the film, showed the surface to be composed of PMMA domains (green) embedded within the PS matrix (red) while STXM, which operates in transmission mode, clearly showed layers of PMMA domains at different depths (dark green and light green) within the PS matrix (Figure 2b). Even after annealing, the PMMA domains and PS matrix do not fully extend through the full thickness of the ~100 nm film, which adds greater complexity to the STXM analysis. Thus, for the study of protein interactions with PS/PMMA surface, X-PEEM is more surface sensitive. In later STXM studies with micron-scale phase segregating polymer blends, the films were made with thicknesses in the 20–40 nm range. In such cases the individual components do in fact span the full thickness [28].

**Appendix Figure 1:**
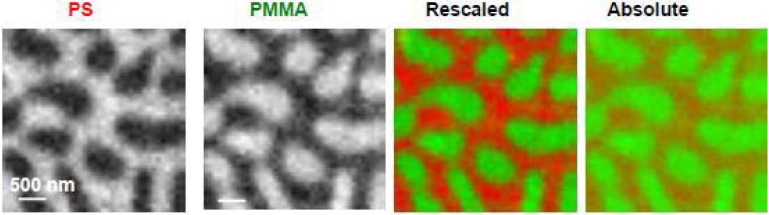
Component maps for PS and PMMA of a PS/PMMA 30/70 blend obtained from STXM. PS color coded in red, PMMA color coded in green. Rescaled image shows phase segregation of the two polymers. Absolute image shows that there is a layer of PMMA all across the sample, which is not from intermixing, but rather from segregation of the PMMA to the mica substrate on which it was spun cast. X-PEEM studies of that surface (results not shown) also show a PMMA rich coating over the bulk PS/PMMA domain pattern.

The C 1s NEXAFS spectra of PS and PMMA are easily distinguishable with PS dominated by a transition at 285.15 eV which arises from the C1s → π*_C=C_ of the benzene ring while PMMA shows a characteristic transition at 288.50 eV, which corresponds to the C1s → π*_C=O_ of the ester group (Figure 2c). Quantitative analysis obtained by fitting the experimental average NEXAFS spectrum with PS and PMMA reference spectra revealed the surface area to be composed of 62(5)%/38(5)% for the PS/PMMA 30/70 film. Strikingly, the surface was dominated by PS, although the majority component of the starting solution was PMMA. This material thus reveals significant differences between the surface and the bulk. STXM imaging through the entire film thickness showed that a larger amount of PMMA was present below the 10 nm depth, and in the bulk PMMA likely forms the continuous phase. Furthermore, recent STXM results for PS/PMMA 30/70 suggest that a PMMA-rich layer exists closest to the polar silicon nitride surface (Appendix Figure 1).

##### 3.1.1.2. Fibrinogen (Fg) Adsorption to PS-PMMA—Buffer *vs.* Water

Two plasma proteins were used for adsorption studies: human fibrinogen (Fg) and human serum albumin (HSA). The NEXAFS spectra of Fg and HSA are similar [59]. The C 1s spectrum is characterized by a small transition at 285.15 eV arising from the contribution of aromatic amino acid residues and an intense transition at 288.20 which corresponds to the C1s → π*_CONH_ transition of the peptide bond. The amide π* feature is 0.3 eV lower than the C1s → π*_C = O_ of PMMA, which occurs at 288.45 eV. With careful energy calibration, the three components (PS, PMMA, and protein) can be easily identified.

**Figure 3:**
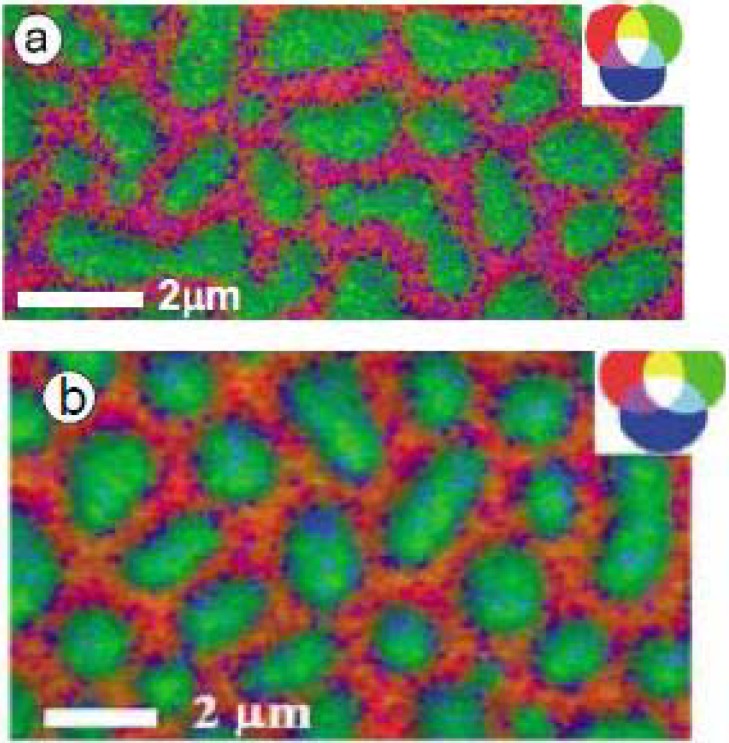
Color coded rescaled component maps of 0.05 mg/mL Fg adsorbed to PS/PMMA from (a) buffer and (b) water. PS = red, PMMA = green, Fg = blue. [26] (reproduced with permission of the publisher).

Fg (0.05 mg/mL) was adsorbed from phosphate buffer (10 mM) to PS/PMMA [26]. Figure 3a shows the X-PEEM absolute qualitative mapping of this system with PS, PMMA and Fg color coded in red, green and blue, respectively. The PS continuous phase is strongly pink arising from the combination of “red” PS and “blue” Fg, while in contrast, the domains of PMMA remain almost pure green, showing a greater thickness of Fg adsorbed on the PS domains. The quantitative results support these qualitative observations with 19(5)% and 9(5)% Fg detected on the PS-rich and PMMA-rich regions, respectively.

In comparison, the absolute qualitative mapping of Fg adsorbed from 0.05 mg/mL solution in doubly distilled deionized (DDI) water to PS/PMMA showed the PS domains to be essentially red (Figure 3b). The quantitative analysis showed 0(5)% Fg adsorbed to PS and 19(5)% adsorbed to PMMA, essentially a reversal compared to the buffered system. As the qualitative maps show, in each case, most of the protein adsorbs to the interface between the PS and PMMA domains. These results were supported by data on ^125^I radiolabelled Fg adsorption to pure PS, pure PMMA and the PS/PMMA blend. For the buffer system, at almost all Fg concentrations sampled (0.0–1.0 mg/mL Fg) adsorption to PS was about 2-fold greater than on PMMA. For adsorption from DDI water, very similar adsorption isotherms were seen over the range of 0.0–0.5 mg/mL and almost no preference was observed [26].

##### 3.1.1.3. HSA adsorption to PS-PMMA

**Figure 4:**
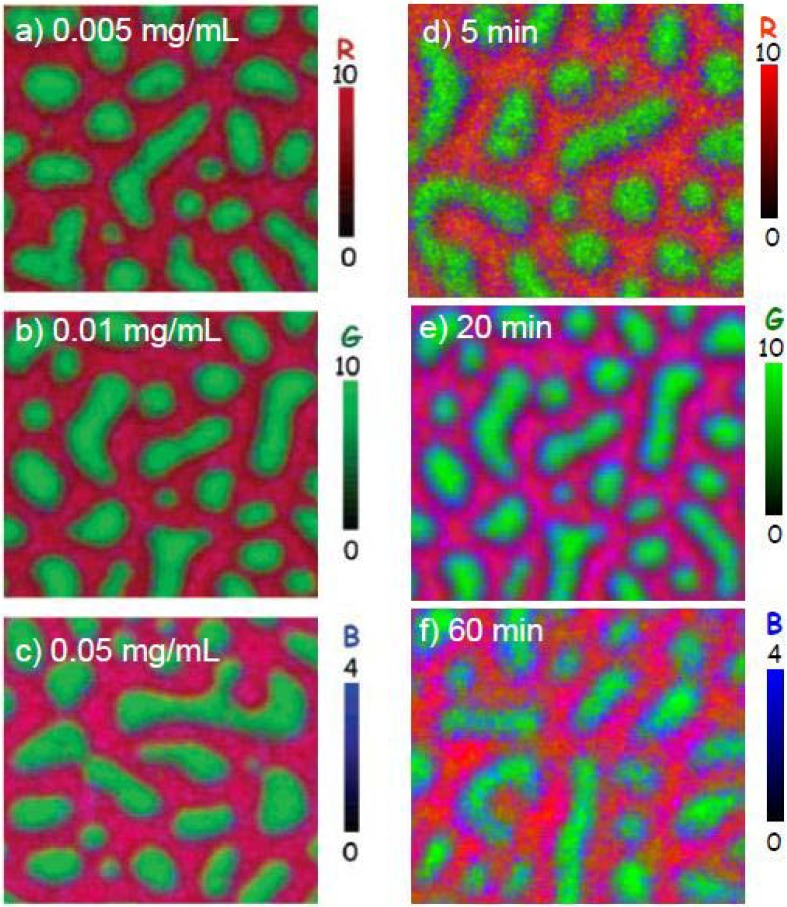
X-PEEM color coded composite maps of HSA adsorbed to PS/PMMA films. (Left panels) HSA concentration dependence: (a) 0.005 mg/mL, (b) 0.01 mg/mL, (c) 0.05 mg/mL in each case for 20 minutes exposure time prior to rinsing; (Right panels) Exposure time dependence: 0.01 mg/mL HSA adsorbed to PS/PMMA for: (d) 5 min, (e) 20 min, (f) 60 min. PS = red, PMMA = green, HSA = blue [28] (reproduced with permission of the publisher).

The surface of a 30:70 PS/PMMA blend was exposed to HSA at concentrations of 0.005, 0.01 and 0.05 mg/mL for 20 min from DDI water [28]. The absolute X-PEEM color-coded images are presented in Figure 4a-c. At low HSA concentrations (0.005 mg/mL), the PS and PMMA domains are red and green, respectively; however, at higher HSA concentrations (0.05 mg/mL), the PS and PMMA regions are pink and teal, signifying that more HSA adsorbs to the surface as the protein concentration increases.

The quantitative results clearly show that adsorption increases from interface (2.5–2.8 nm of adsorbed HSA) > PS (2.1–2.6 nm of adsorbed HSA) > PMMA (1.3–2.1 nm of adsorbed HSA). This trend is slightly different compared to the one observed for Fg adsorption to PS/PMMA, where Fg prefers the less hydrophobic surface of PMMA when adsorbed from DDI. Since the physiological ionic strength of plasma is ~150 mM [60], we suggest the preference of Fg for PMMA under zero ionic strength may result from adsorption via a non-native protein conformation, where the more polar amino acid residues may preferentially adsorb to the polar PMMA domains.

The HSA adsorption to PS/PMMA was also studied with respect to exposure time. HSA (at 0.01 mg/mL concentration) was adsorbed to PS/PMMA for 5, 20 and 60 min. The rescaled color-coded maps show that HSA adsorption to the surface is varied, depending on exposure times. With a 5 min exposure, the PS and PMMA domains are more red and green, respectively, and become pinker and more teal as time increases to 20 and 60 min exposures (Figure 4d-e).

Similar HSA thicknesses were observed for the PS-rich region, regardless of exposure time, while 0.4(5) nm more HSA was adsorbed at the interface at shorter exposures (3.0(5) nm) compared to longer exposures (2.6(5) nm). In contrast, the amount of HSA adsorbed to the PMMA region increased significantly from 0.2(5) nm to 1.9(5) nm as the time increased from 5 to 60 minutes, respectively.

Clearly, this study showed that at low HSA concentration and short exposure times, HSA prefers the inter-domainal regions between PS and PMMA. At longer exposure times, there is evidence of redistribution of protein towards mainly the PMMA region and to some extent, the PS region. Since HSA is a globular protein with both exposed hydrophobic and hydrophilic domains, it is suggested that the hydrophobic areas bind more tightly to PS (contact angle of 97°) and the hydrophilic regions prefer PMMA (contact angle 76° [61]). Moreover, since the PS matrix is favored initially over PMMA, hydrophobic interactions are certainly involved in the adsorption process.

**Figure 5:**
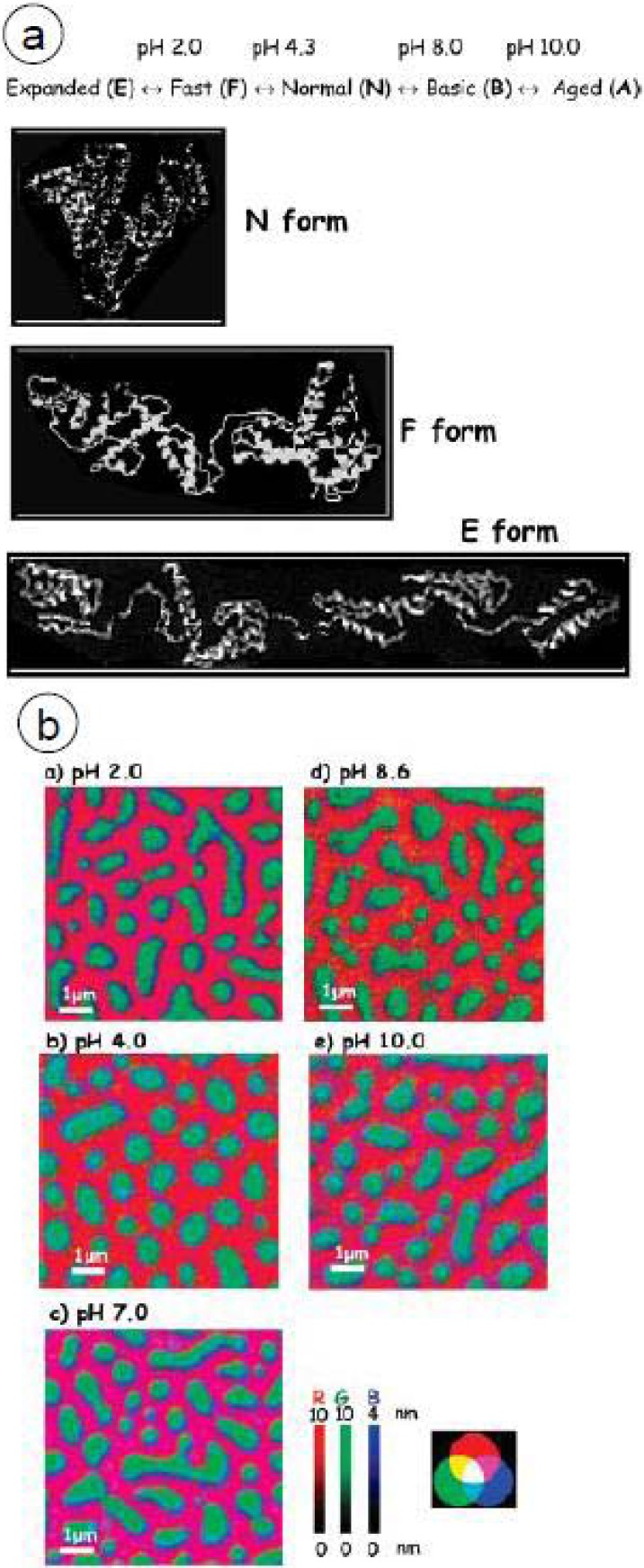
(a) Conformation of HSA at various pH and (b) Rescaled color coded images of HSA adsorbed to PS/PMMA at the C 1s edge at five different pH. PS = red, PMMA = green and HSA = blue. [29] (reproduced with permission of the publisher)

Furthermore, 0.05 mg/mL HSA was adsorbed to the PS/PMMA surface at pH 2.0, 4.0, 7.0, 8.6 and 10.0 [29]. In aqueous solution, HSA exists in five pH-dependent conformations: E, F, N, B, and A (Figure 5a) [62]. At lower pH, HSA exists in an unfolded and expanded conformation, while at higher pH HSA is compact. The isoelectric point of HSA is 4.7–5.4, with a charge of almost -30 at pH 8.8 [63].

Figure 5b shows the rescaled color-coded maps of HSA adsorbed to PS/PMMA with PS, PMMA and HSA shown in red, green and blue, respectively. The wide variation in color reveals differences in the adsorption distribution with respect to pH and protein conformation. Strikingly, the blue color corresponding to HSA is consistently strongest at the PS/PMMA interface regardless of pH. This interfacial area is likely the area of the lowest free energy. At extremely alkaline and basic pH, similar amounts of HSA are adsorbed to the PS and PMMA domains. However, at pH 4, close to the isoelectric point, there is a preference for PMMA over PS, while at pH 8.6 there is a preference for PS over PMMA.

Since the HSA conformation varies depending on pH, it is possible to qualitatively interpret the X-PEEM data in terms of the amounts of hydrophobic and hydrophilic regions present at the protein surface at different pH by analyzing the amount of protein adsorbed to different areas of the surface. For instance, at pH 8.6, as the conformation of HSA contracts, more hydrophobic residues may be present at the surface, thus resulting in an adsorption preference for hydrophobic PS. In contrast, as the conformation of HSA expands at pH 4, possibly more hydrophilic residues are present, giving rise to a greater amount of protein adsorbed to the polar PMMA region.

##### 3.1.1.4. Protein-peptide competitive adsorption

While distinguishing one protein from another protein is difficult with NEXAFS spectroscopy due to spectral averaging over relatively similar distributions of amino acids [64], peptides with an usual amino acid sequence may exhibit a NEXAFS spectrum different from proteins in general. The peptide SUB-6 (RWWKIWVIRWWR-NH2), an arginine-rich antimicrobial cationic peptide [65], was found to exhibit a NEXAFS spectrum quite different from HSA, with a characteristic transition at 289.3 eV arising from the guanidine group of arginine. STXM mapping of SUB-6 dusted onto a background of HSA easily distinguished the two bio-components [59].

**Figure 6:**
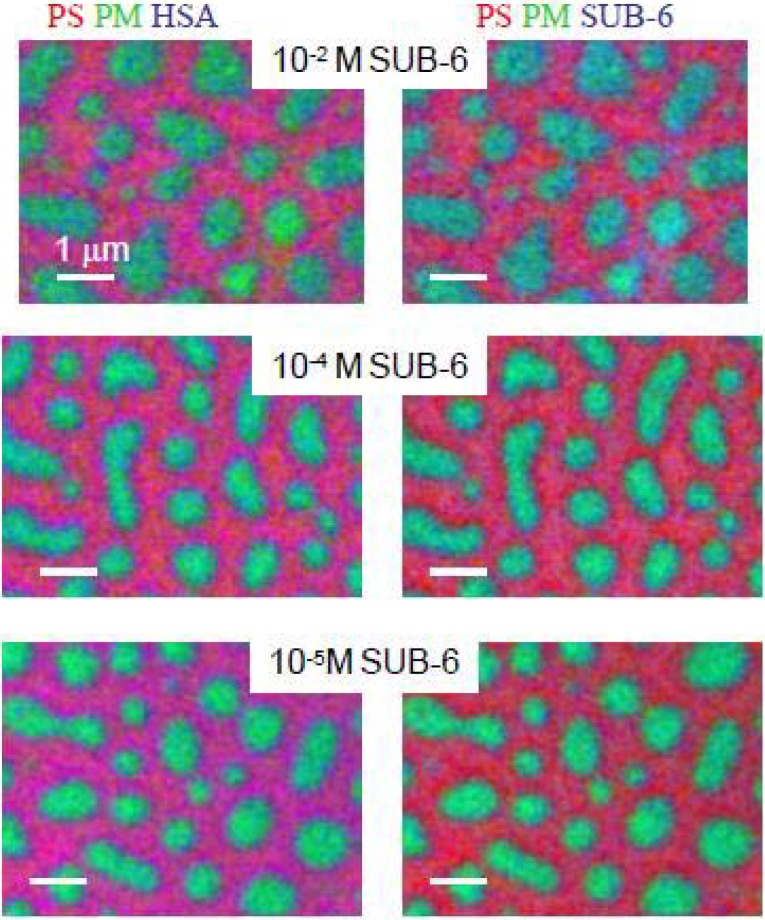
Color-coded rescaled component maps for SUB-6/HSA mix adsorbed to PS/PMMA at SUB-6 concentration (top) 1 × 10^–2^ mg/mL, (middle) 1 × 10^–4^ mg/mL, (bottom) 1 × 10^–5^ mg/mL, in each case, with the. HSA concentration held unchanged at 0.05 mg/mL. PS = red, PMMA = green, HSA = blue, left, SUB-6 = blue, right [30] (reproduced with permission of the publisher).

SUB-6, at concentrations varying from 1 × 10^–2^ to 1 × 10^–5^ mg/mL, was adsorbed to the PS/PMMA surface [30]. As with HSA adsorption to PS/PMMA, SUB-6 adsorbed preferentially to the inter-domainal interface. However, SUB-6 also showed a strong adsorption preference to PMMA over PS. This preference is likely due to attraction of the positive (+5) charge of SUB-6 to the polar PMMA. For the protein-peptide competitive adsorption, the HSA concentration was held constant at 0.05 mg/mL while the SUB-6 concentration was varied from 1 × 10^–2^ to 1 × 10^–5^ mg/mL. The absolute images are presented in Figure 6 with PS color coded in red, PMMA color coded in green and HSA color coded in blue on the left side and SUB-6 color coded in blue on the right side. The shading of red, green and blue for HSA adsorption is similar suggesting that the amount of HSA adsorbed to the surface does not vary greatly, as expected, since the concentration of HSA is held constant. At high concentrations of SUB-6, the peptide adsorbs strongly to the PMMA domains as evidenced by the very blue PMMA domains. Even at lower concentrations (1 × 10^–5^ mg/mL SUB-6), the PMMA domains are markedly more blue for peptide compared to protein.

Comparison of the amount of SUB-6 adsorbed to the PS/PMMA surface in the mixed competitive adsorption with the pure peptide system revealed that more peptide adsorbed to the surface when HSA was present. This synergistic effect was found to occur due to the presence of a protein-peptide complex, formed via an electrostatic attraction between the positively charged peptide and the negatively charged protein.

At pH 12, the positive charge on SUB-6 is mostly neutralized. Co-adsorption of both protein and peptide to the PS/PMMA surface under alkaline conditions (pH 12) revealed redistribution of both components. The thickness of peptide decreased on the PMMA regions and increased on the PS region. HSA adsorption was substantially lower at alkaline pH, which indicates that the PMMA domains were mostly covered by the peptide. Thus, the protein-peptide complex formation was suppressed by moving to more alkaline pH.

#### 3.1.2. Biodegradable or Biocompatible Polymers

##### 3.1.2.1. HSA adsorption to polystyrene-polylacide (PS-PLA)

Polylactide (PLA) is a biodegradable polymer [66,67,68] that has been approved by the US Food and Drug Administration (FDA) [69] for use in biomedical and pharmaceutical applications. Blends of PS and PLA have been investigated as possible materials for packaging and tissue engineering [70].

Blends of PS and PLA were studied in our lab with respect to blend composition, total polymer concentration in the solution, and temperature [31]. For all polymer compositions studied, AFM images showed that one or other polymer dominated the surface. From X-PEEM analysis of PS:PLA 40:60 and 60:40, this surface enrichment was shown to correspond to PLA (60–92% enrichment in the top 10 nm of the surface). Even dilution from 1 wt % polymer to 0.7 wt % polymer for the PS:PLA 60:40 blend did not significantly change the surface composition.

However, annealing above the glass transition temperature (T_g_) of PLA (T_g_ ~ 60 °C for PLA) revealed significant changes to the surface morphology. The AFM imaging of the 0.7 wt % PS:PLA 60:40 blend annealed for 1 h at 70 °C showed significant coarsening of the domains and an increase in the height of the domains from 35 nm (un-annealed) to 95 nm (annealed). X-PEEM analysis of the blend surface showed a surprising phase inversion where prior to annealing, PLA domains were embedded in a PS matrix, and after annealing, the PS formed domains within a PLA continuous matrix. This phase inversion was proposed to occur due to the thermal evolution of polymer viscosities since the parameters affecting phase inversion (such as volume fraction, tension, shear stress and strain) remained relatively unchanged [71,72]. Nonetheless, by annealing at 70 °C, we were able to obtain a highly phase segregated PS-PLA surface, with 90% PS dominating the PS-rich regions and 77% PLA in the PLA-rich regions.

**Figure 7:**
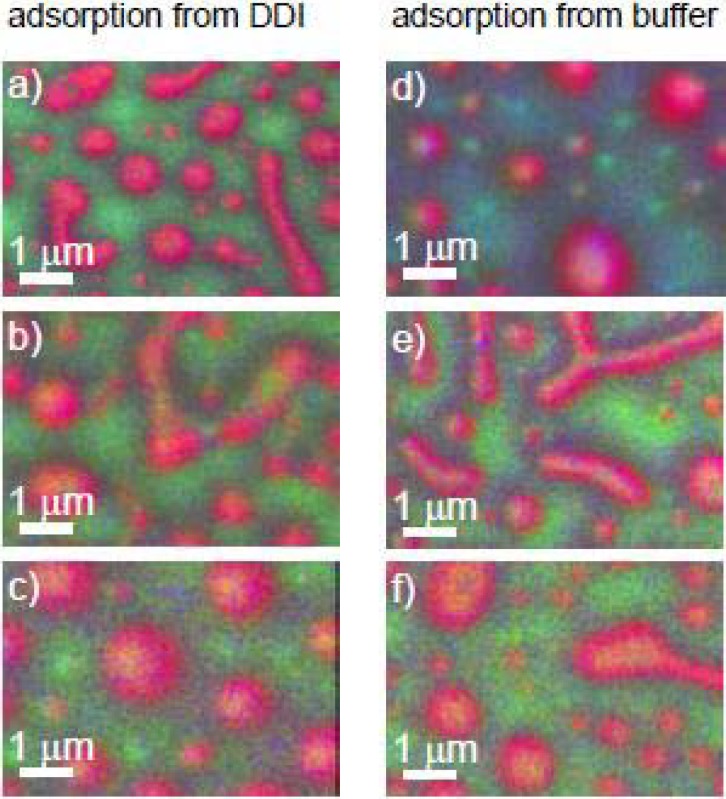
X-PEEM color coded composite maps of 40:60 PS/PLA films (0.7 wt % loading) annealed 1 h at 70 °C with HSA adsorption from water: (a) 0.05 mg/mL, (b) 0.01 mg/mL, (c) 0.005 mg/mL; HSA adsorption from buffer: (d) 0.05 mg/mL, (e) 0.01 mg/mL, (f) 0.005 mg/mL. PS = red, PLA = green, HSA = blue [32] (reproduced with permission of the publisher).

HSA was adsorbed to the 0.7 wt % PS:PLA 60:40 surface annealed for 1 h at 70 °C at three different concentrations (0.005, 0.01 and 0.05 mg/mL) from both buffer and DDI water [32]. Figure 7a shows the absolute color-coded map of HSA adsorption from DDI at high protein concentration (0.05 mg/mL) with PS, PLA and HSA in red, green and blue, respectively. The map is strongly pink and teal, indicative of protein adsorption to the surface. As the concentration of HSA decreases, the images become redder and more pure green pixels are evident, showing that there is less uniform protein coverage at lower protein concentration (0.005 mg/mL) (Figure 7a-c). In contrast, the absolute image for HSA adsorption to the surface from PBS buffer at 0.05 mg/mL reveals a very blue and pink map. Here, a significantly greater thickness of HSA is detected compared to adsorption from DDI at the same protein concentration. As the concentration of protein decreases, the maps become comparable between DDI and buffer (Figure 7d-e).

The quantitative results show that the detected thickness of HSA resulting from adsorption from a PBS solution is almost twice as great as the thickness of HSA resulting from adsorption from DDI [32]. X-PEEM shows that as the ionic strength increases, there is a general increase in protein thickness, which conflicts with literature reports of a decreased number of adsorbed protein molecules with respect to increasing ionic strength [73,74,75]. However, according to integrated optical methods, although the number of adsorbed protein molecules decreased with ionic strength, the area occupied by the adsorbed molecules increased almost 10-fold at NaCl concentrations of 0.5 mol/L. ^125^I radiolabeled HSA was used to verify if X-PEEM was detecting a greater number or greater area of adsorbed protein molecules. Radiolabeling revealed that at higher HSA concentrations (0.05 and 0.01 mg/mL) the number of adsorbed HSA molecules from DDI water were almost double that compared to buffer, while similar numbers of protein molecules were adsorbed at lower concentration (0.005 mg/mL). Thus X-PEEM also provides an indirect method of assessing protein conformation, since a larger conformation must be adsorbing to the surface under buffer conditions to account for the greater detected protein thickness.

**Figure 8:**
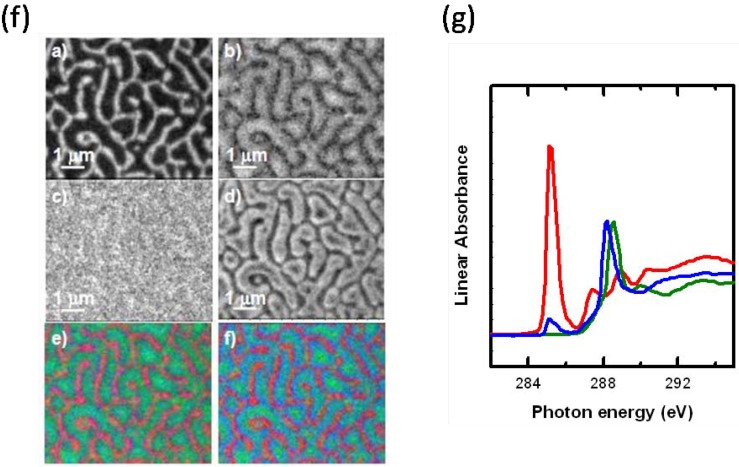
Component maps measured from a 40:60 PS:PLA ratio annealed 6 h at 45 *°*C. Component maps of a) PS), b) PLA, c) HSA, derived from a C 1s image sequence. d) HSA component map obtained from fit to the N 1s image sequence of the same area. e) Rescaled color composite map based on component maps derived from the C 1s image sequence (red = PS, green = PLA, blue = HSA). f) Rescaled color composite map combining maps from C 1s and N 1s (red = PS (C1s), green = PLA (C1s), blue = HSA (N1s)). G) C1s NEXAFS spectra of PS (red), PLA (green) and HSA (blue). [32] (reproduced with permission of the publisher).

Another advantage of X-PEEM is the use of multi-edge fitting to obtain greater precision in locating the preferred areas of protein adsorption. Since HSA is the only component in our system to contain nitrogen, by combining the C1s map of PS and PLA with the N1s map of HSA in the exact same region, a more sensitive map is obtained (Figure 8). Here, the map from the multi-edge fitting shows much more clearly that the interface is the preferred region of HSA adsorption.

##### 3.1.2.2. HSA adsorption to PMMA-chitosan blends

Chitin, or 2-acetamido-2-deoxy-β-D-glucose, is a natural polysaccharide found in the shell of crustaceans or insects [76]. The N-deacetylated derivative is chitosan. Chitosan is biodegradable and nontoxic, and is under consideration as a biomaterial for a variety of applications [76].

**Figure 9:**
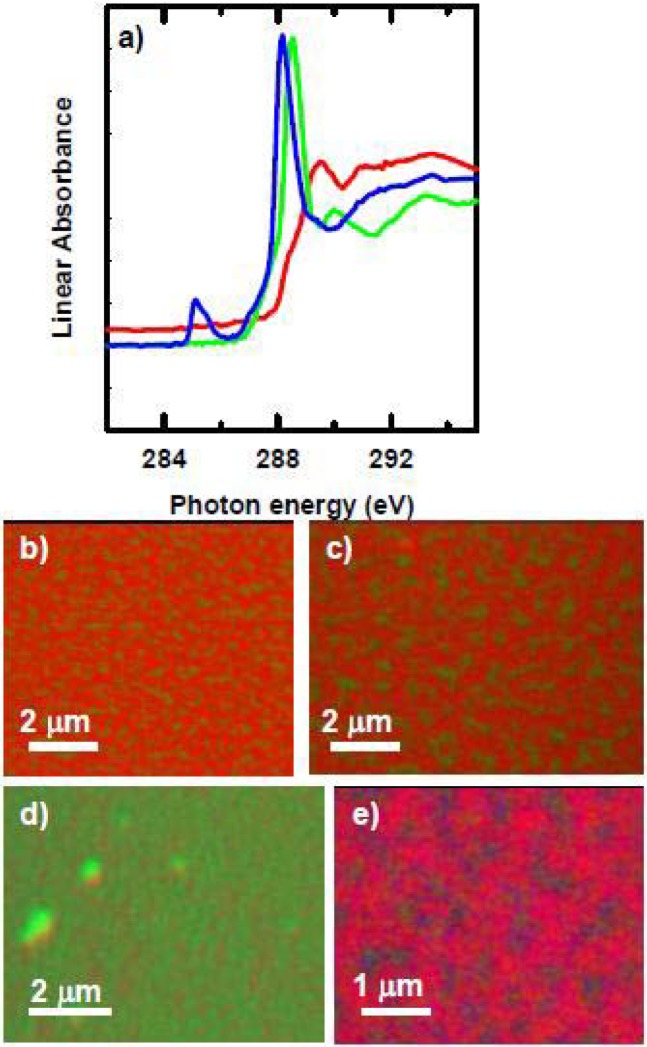
(a) Reference spectra of Chitosan, PMMA and HSA, (b) Chitosan/PMMA 60:40 unannealed, (c) Chitosan/PMMA annealed 18 h at 160 °C, (d) Chitosan/PMMA 40:60 annealed 18 h at 160 °C, (e) 0.05 mg/mL HSA adsorbed to annealed Chitosan/PMMA 60:40 for 20 min. Chitosan = red, PMMA = green, HSA = blue.

The NEXAFS spectrum of chitosan shows an intense transition at 289.6 eV, which is similar to the C1s → σ*_C-OH_ transition assigned for arabinose and rhamnose at 289.44 eV [77], and is easily distinguished from the PMMA C1s → π*_C=O_ at 288.5 eV (Figure 9a).

Chitosan was blended with PMMA in a co-solvent of glacial acetic acid and water and then spun cast onto Si(111). Figure 9b shows the color-coded X-PEEM component maps for the blends, with chitosan and PMMA color-coded as red and green, respectively. The absolute image of chitosan:PMMA 60:40 is intensely red, revealing that surface segregation of chitosan occurs. From the quantitative analysis, there is ~86% chitosan in the chitosan-rich regions, and ~68% chitosan in the PMMA rich regions. Annealing for 18 h at 180 °C, above the Tg of PMMA did not significantly change the surface composition, although the PMMA domains coalesced and grew larger (Figure 9c). Changing the mass ratio to chitosan:PMMA 40:60 (and annealing for 18 h at 180 °C) significantly changed the surface: PMMA dominated both the PMMA-rich (83% PMMA) and chitosan-rich (61% PMMA) regions (Figure 9d).

Figure 9e shows the color composite map for 0.05 mg/mL HSA adsorption to the chitosan:PMMA 60:40 annealed surface. However, it was difficult to isolate specific pixels corresponding to the interface, PMMA-rich and chitosan-rich regions since the size of the phase segregated domains is close to the spatial resolution of the microscope. Nonetheless, the qualitative map shows significant protein adsorption, as indicated by the strong pink and blue colors.

##### 3.1.2.3. HSA adsorption to PEO-PETA blends

Polyethylene oxide (PEO) is a hydrophilic polymer used to reduce protein adsorption [78] or improve biocompatibility [79]. Since PEO is hydrophilic, techniques such as γ [80], UV [81,82] and electron irradiation [83] have been used to crosslink the PEO chains to prevent dissolution upon protein exposure. In particular, UV-initiated crosslinking of PEO with radical crosslinkers such as pentaerythritol triacrylate (PETA) [84,85] is becoming increasingly common for biomedical applications such as drug delivery [86,87] or to chemically pattern surfaces for cell studies [88].

PEO was blended with 1.5, 5 and 10 wt % PETA and spun cast onto Si(111) at 4000 rpm for 40 s. The films were then exposed to 0.05 mg/mL HSA for 20 min before vigorous washing and analysis with X-PEEM [33]. This study found that PETA is a surface active molecule. As the concentration of PETA increased from 1.5 to 10 wt %, the composition of the top 10 nm of the surface became dominated by PETA. At 1.5 wt % PETA, approximately 10(5)% PETA was detected at the surface. However, as the concentration of PETA increased to 5 wt % and 10 wt %, the surface was composed of 45(5)% and 68(5)% PETA, respectively.

Furthermore, a marked correlation was seen with increasing PETA concentration and increasing HSA adsorption. At 1.5 wt % PETA, no adsorbed HSA was detected via X-PEEM. While at 5 wt % and 10 wt % PETA, 0.3–0.7(5) nm and 1.3–1.4(5) nm of HSA, respectively, was adsorbed to the surface, showing that above PETA concentrations of 5 wt %, PEO begins to lose its biocompatibility. Thus, this investigation on the effect of PETA crosslinked PEO revealed that when PETA is present as major component at the surface of PEO films, the antifouling properties of PEO are compromised.

##### 3.1.2.4. Fluoropolymers for stent coating applications (Substrate characterization)

Hydrophobic fluorocarbon coatings for vascular stents, (metallic medical devices used to open arteries) have been proposed to prevent release of transition metal elements such as nickel, molybdenum and chromium present in stainless steel since these species can cause an inflammatory response upon elution into the body [89]. The perfection of such coatings is critical to their function, and this aspect was investigated by X-PEEM [34]. A fluoro-polymer was plasma deposited on disk-shaped stainless steel substrates to produce thin and ultra-thin samples of 100 and 35 nm thickness, respectively [90]. Next the coated samples were plastically deformed at 25% using a testing machine to simulate the mechanical stress they would experience in the body. X-PEEM was then used to image the surface distribution of Cr, Fe, C and F across the ultra-thin (35 nm thick) fluoro-polymer surface before and after deformation. Prior to deformation, the surface was flat with infrequent defects; however, after 25% deformation large surface imperfections appeared along grain boundaries and slip bands. For this sample, Fe and Cr contributions at the surface were detected after deformation. Although evidence of transition metals were detected, the fissures and defects in the coating were below the spatial resolution of the X-PEEM microscope but nano-pinholes were detected with scanning electron microscopy (SEM).

The goal of this study was to fabricate a pinhole-free fluorocarbon polymer coating to increase the biocompatibility of stents. The aim of the coating is to prevent corrosion and to store and release drug molecules. X-PEEM (combined with other surface sensitive techniques such as XPS and TOF-SIMS) was successfully used to characterize the coating surface. The results from this study showed that further optimization is required to obtain coatings that follow FDA regulations.

#### 3.1.3. Protein adsorption under hydrated conditions

##### 3.1.3.1. Fg adsorption to poly(styrenecoacrylonitrile) (SAN) and poly-isocyanate poly-addition product (PIPA) nanoparticles embedded in polyurethane

**Figure 10:**
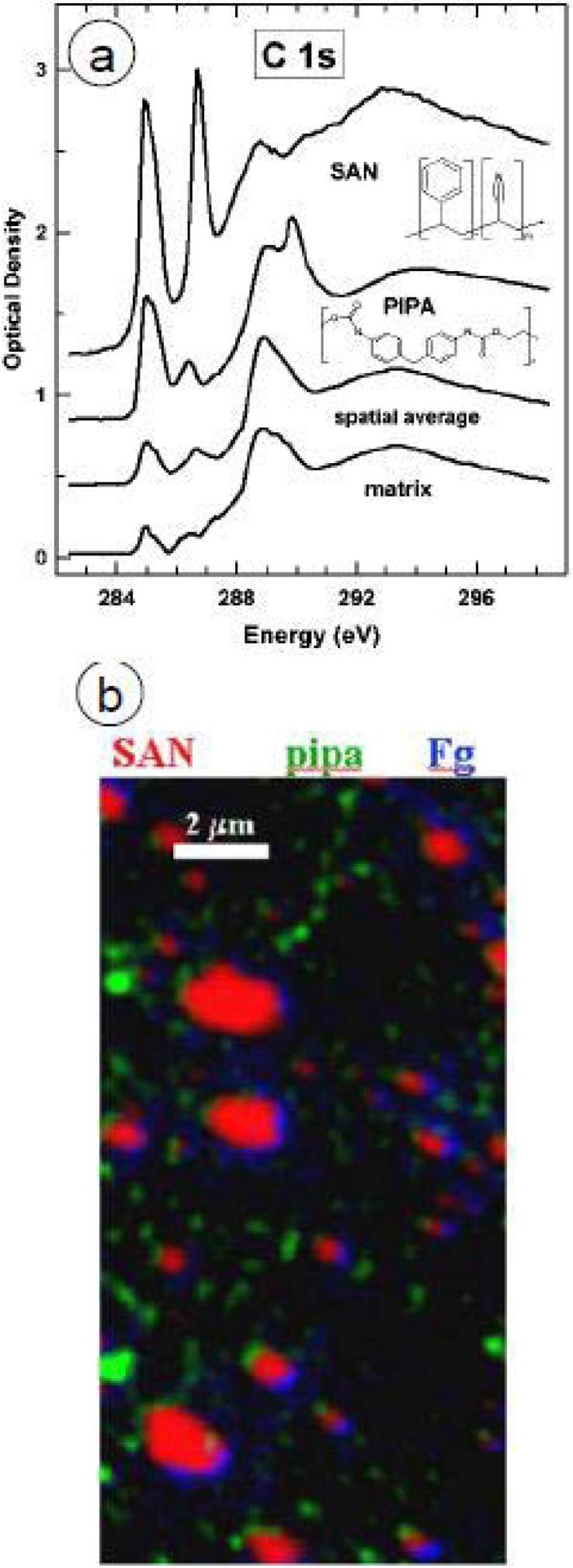
(top) C 1s spectra of polyurethane matrix, PIPA and SAN components of a phase segregated mixed-filler polyurethane film. (bottom) Rescaled color coded composite map (red = SAN, green = PPIA, blue = HSA. [91]. (reproduced with permission of the publisher).

Microtomed thin sections of polyurethane embedded with poly(styrene-co-acrylonitrile) (SAN) and poly-isocyanate poly-addition product (PIPA) nanoparticles were examined with STXM, and the imaging revealed the SAN and PIPA nanoparticles to be ~ 0.1 – 2 μm in diameter [91]. The NEXAFS spectra of both PIPA and SAN have an intense peak at 285.2 eV, arising from the C 1s → π*_C=C_ transition of the phenyl rings (Figure 10a) [92]. SAN has an additional feature at 286.7 eV corresponding to the C1s → π*_C≡N_ transition, while PIPA has a sharp C 1s → π*_C=O_ feature at 289.9 eV, both of which are useful for distinguishing SAN from PIPA. The polyurethane matrix is mainly polyether which exhibits a broad peak at ~289 eV from C1s → σ*_C-O_ transition of the polyether chains.

Fg (0.1 mg/mL) was adsorbed to the SAN/PIPA/polyurethane surface for 20 min and then rinsed vigorously and air dried. Figure 10b presents the color-coded map, with SAN, PIPA, Fg, and polyurethane in red, green, blue and black respectively. Fg strongly prefers to adsorb to the interface between SAN and the polyurethane matrix, with a larger amount of the Fg on the matrix side of the interface. Furthermore, a second sample with dried Fg adsorbed to the surface was re-hydrated with a drop of de-ionized water and sandwiched between two silicon nitride windows, with the edges sealed with epoxy, forming a hydrated wet cell. The quantitative analysis shows that under hydrated conditions, Fg also prefers to adsorb to the interface between SAN and matrix, with approximately 10 nm of Fg on the SAN particles, but over 60 nm of Fg adsorbed at the interface between SAN and polyurethane. The preference for the matrix over SAN is also evident under hydrated conditions. Since human Fg has dimensions of ~ 45 × 9 × 6 nm [93], as determined by electron microscopy, at the interface Fg may be adsorbing end-on rather than side-on.

Although the exact mechanism of Fg adsorption to the interface is not clear, it may be due to the surface topography since the SAN particles protrude up to 50 nm from the surface. Rechendorff *et al*. have found a correlation between surface roughness and increased Fg adsorption [94]. This may result from the ability of Fg to adsorb anisotropically with different orientations, thus forming additional bonds to both the polyurethane surface and the SAN particles [35].

##### 3.1.3.2. HSA adsorption to PS/PMMA

**Figure 11:**
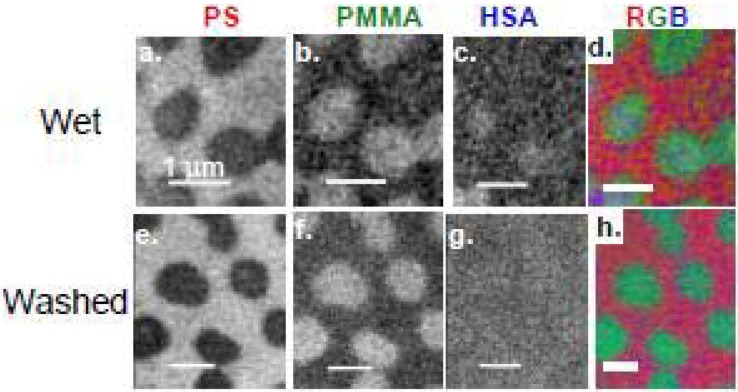
(top) Component maps derived from a C 1s STXM image sequence: fully hydrated wet cell of HSA adsorbed to a PS-PMMA thin film. a) PS b) PMMA, c) HSA, d) rescaled color composite map. (bottom) Component maps from a fully hydrated sample, but washed repeatedly with DDI water prior to sealing the wet cell. e) PS, f) PMMA, g) HSA, h) rescaled color composite map. In each case, the color coding for the composite maps is (red = PS, green = PMMA, blue = HSA). HSA = [0.005 mg/mL]. [36]. (reproduced with permission of the publisher).

HSA (0.005 mg/mL, 2 μL) was adsorbed to PS/PMMA, and enclosed within a wet cell of two Si_3_N_4_ windows for STXM analysis. The component maps of PS, PMMA and HSA are shown in Figure 11a-c with the color coded map shown in Figure 11d with PS, PMMA and HSA color coded as red, green and blue, respectively [36]. Close inspection of the HSA component map reveals a direct correlation between the bright white pixels of HSA and the PMMA domains. The quantitative results reveal ~10 nm of HSA adsorbed to the PMMA-rich areas, which suggests an end-on type adsorption, since the crystallographic dimensions of HSA are 8 × 8 × 3 nm [95]. However since both surface plasmon resonance (SPR) [5] and neutron reflection [96] data for hydrated HSA and bovine serum albumin (BSA) adsorption to PS and silica suggest side-on adsorption, it is possible that STXM is sampling a bilayer of adsorbed HSA molecules.

HSA (0.005 mg/mL, 2 μL) was adsorbed onto a PS/PMMA substrate, vigorously washed off after 20 min, and the resulting 2 μL of DDI water left was sandwiched between two Si_3_N_4_ windows to keep the adsorbed protein hydrated (Figure 11 h). The quantitative results show similar thicknesses of HSA adsorbed to PS in both the washed and unwashed case, while the thickness of HSA adsorbed to PMMA in the washed system is 50% less (5.6 nm) compared to the unwashed adsorption. This reveals that a large proportion of the <10 nm thick adsorbed HSA is likely due to loosely bound protein which can be removed by washing.

##### 3.1.3.3. Electrolyte-induced deswelling behavior of pH-responsive microgels

pH-responsive hydrogel nanoparticles (microgels) have many potential applications including water purification [97,98], sensors [99] and drug delivery vehicles [100,101,102]. Poly(2-vinylpyridine) nanoparticles exhibit swelling behavior at low pH (~ 4) due to protonation of the pyridine residues resulting in cationic repulsion of the charged P2VP chains. This degree of swelling is suppressed at high salt concentration (up to 2.0 M NaCl). P2VP latex/microgel particles were prepared by emulsion polymerization of 2VP and divinylbenzene in aqueous solution. Poly(ethylene glycol) methacrylate (PEGMA) was then grafted to the outside of the particles to act as a steric stabilizer [37]. The microparticles were sandwiched between two Si_3_N_4_ windows to maintain hydration during imaging. STXM was successful in monitoring the swellability of the P2VP microparticles as a function of pH and salt concentration. Furthermore, N1s NEXAFS spectroscopy of the nanoparticles revealed differences in the spectroscopy between neutral and charged particles with the N1s to π* transition (298.9 eV) of the neutral particles 1.5 eV lower than the π* transition for the cationic particles. This study is the first direct visualization of the effect of electrolyte on the swellability of hydrogel nanoparticles under aqueous conditions. Furthermore, STXM X-ray spectromicroscopy can be used to provide useful quantitative chemical information, such as the degree of protonation.

## 4. Summary and Conclusions

Our laboratory has investigated protein adsorption to heterogeneous surfaces using soft X-ray spectromicroscopy for over a decade. X-PEEM and STXM imaging combined with NEXAFS spectroscopy is one of the few chemically sensitive tools available for the study of spatial distributions in protein adsorption to polymeric surfaces, with a spatial resolution in the 50–80 nm range.

We found that protein adsorption occurs primarily at the inter-domainal interfaces of polymer blend surfaces. Furthermore, protein adsorbs to hydrophobic domains in preference to hydrophilic ones. Hydrophilic PEO showed strong protein resistance in agreement with many other recent reports. When protein adsorption was investigated under hydrated conditions, protein conformational changes were observed between wet and dry conditions [36]. FTIR spectroscopy in the amide I region has shown that structural distortions result from protein dehydration [103]. In fact, it was found that the β-sheet content increased by 16% and the α-helix decreased by 28% upon lyophilization [104]. Although these experiments refer to freeze-dried proteins, it is possible that similar structural rearrangements occur from air-drying adsorbed proteins.

While this review discusses many advantages of X-ray spectromicroscopy, there are also several disadvantages. In situ biological experiments using X-PEEM and STXM are problematic due to the limited physical space between the microscope optics and the sample, and the helium or UHV sample chamber requirements. Furthermore, both X-PEEM and STXM require ultrathin samples, which may not be easily adapted for imaging and spectral analysis in certain biomaterials applications. The limited NEXAFS spectral contrast between different proteins is currently a major drawback in exploring multi-protein systems, although this may be overcome by introducing a label (although that may alter the adsorption behaviour). Finally, access to synchrotron-based technologies is not readily available for many academic or industrial laboratories.

Nonetheless, soft X-ray spectromicroscopy techniques are emerging as powerful tools for biomaterials analysis. With the implementation of new X-PEEM (aberration correction) and STXM (better zone plates) optics, the microscopes will achieve a spatial resolution, most likely reaching well below 10 nm in the next few years, At this scale the techniques will be able to image individual proteins. As chemical and biological sensors become more compact, X-ray spectromicroscopy techniques will be an advantageous tool for localizing bio-components relative to a patterned substrate (*i.e*., protein microarrays, bacterial biosensors, *etc*.). Furthermore, the development of STXM flow cells will enable time resolved, hydrated protein adsorption experiments.

The ability of soft X-ray spectromicroscopy techniques for mapping and quantitative chemical characterization of biologically relevant surfaces and subsequent protein adsorption to assess biocompatibility has lead to the development and optimization of a powerful analytical tool. With the emergence of hydrated imaging, systems that more closely model *in vivo* conditions can be probed. These techniques are becoming more widely available, with peer review access to PEEM and STXM microscopes available at many synchrotron facilities around the world.
